# Metastasis of Dermatofibrosarcoma from the Abdominal Wall to the Thyroid Gland: Case Report

**DOI:** 10.1155/2012/659654

**Published:** 2012-10-24

**Authors:** Alexander Kreze, Andrea Zápotocká, Tomáš Urbanec, Jiří Koskuba, Mikuláš Pura, Pavel Vítek, Pavol Praženica, Eva Traboulsi

**Affiliations:** ^1^2nd Department of Internal Medicine, Faculty Hospital Bulovka, 18081 Prague, Czech Republic; ^2^National Institute of Endocrinology and Diabetology, 03491 L'ubochňa, Slovakia; ^3^Oncological Institute, 18081 Prague, Czech Republic; ^4^Department of Otorhinolaryngology, Central Military Hospital, 16902 Prague, Czech Republic; ^5^Department of Pathology, Central Military Hospital, 16902 Prague, Czech Republic

## Abstract

Metastases in the thyroid gland are very rare. Even the rarer are sarcoma metastases. A 52-year-old woman was referred to our department for evaluation of a nodule in the right lobe of the thyroid gland. She had a history dermatosarcoma of the abdominal wall with known metastasis in the lung. Clinically she had neck pain and worsened swallowing. Objective assessment (ultrasound, computed tomography, and magnetic resonance) indicated a voluminous right lobe nodule with mechanical syndrome, and a fine-needle aspiration biopsy revealed a very suspicious malignant finding. After surgery, the diagnosis was metastasis of dermatofibrosarcoma protuberans. Subsequent treatment was radio- and chemotherapy.

## 1. Introduction

Thyroid gland tumors account for 0.5–1% of all malignancies [1]. According to the World Health Organisation (WHO) primary thyroid tumors are classified as epithelial and nonepithelial, benign or malignant, with a separate category for lymphomas and miscellaneous neoplasms [2]. Classification according to the Armed Forces Institute of Pathology (AFIP) gives priority to the cell of origin and incorporates, in each cell type, special tumor types andsubtypes designated as “variants” [3]. Sarcomas according to WHO classification are class V, while for AFIP classification they are A4. 

Dermatofibrosarcoma protuberans (DFSP) is arather uncommon soft tissue tumor of mesenchymal origin arising in the dermis. It is regarded as having intermediate malignant potential. DFSP accounts for less than 2% of all soft-tissue sarcomas (less than 0.1% of all cancers). The most common location is the trunk (50%), proximal extremities (30%), head and neck (0%–15%), and rare cases involving the hands and feet. The usual presentation is an asymptomatic firm plaque that may be red, brown, violaceous, or flesh colored. Lesion size ranges from 1 to 5 cm [4]. Histological evaluations show a dense array of spindle-shaped tumor cells, slender nuclei, intracellular collagen deposition, and small capillary blood vessels throughout. Metastasis is quite unusual [4], the majority of soft tissue sarcomas metastasize to the lungs. Less frequently reported sites include the retroperitoneum, mediastinum, bones, and rarely the kidney, brain, omentum, scalp, ovaries, liver, and heart [5]. The first case of DFSP metastasis to the thyroid gland was described by Onoda in 1990 [6]. 

Solitary thyroid nodules are common in clinical practice but nodular intrathyroid metastasis is rare and may be underestimated [7].

The prevalence of intrathyroid metastases of nonthyroid origin ranges from 1.9% to 25% [8].

Direct extension into the thyroid may occur in carcinomas of the pharynx, larynx, trachea, and esophagus. Retrograde lymphatic spread into the thyroid has been reported with breast carcinoma as well as hematogeneous metastases to the thyroid, particularly of malignant melanoma, lung, gastrointestinal, breast, and renal cell carcinomas. Rare sources of primary tumors are choriocarcinoma, malignant phylloides tumors, and sarcoma [1].

## 2. Case Report 

A 52-year-old woman with a personal history including several surgeries: an appendectomy long ago, extirpation of dermatofibrosarcoma of the inguinal suprapubic region on the left side in1998, and surgically treated recurrences in 2000, 2001, 2008, and 2009. The last lesion was as large as 8 × 4 cm. Histological findings were fusiforme and mitotic activity. After last surgical treatment was done local external radiotherapy (66 Gy). 

In 2011 she consulted our endocrinology department following the discovery of recent swelling on the right side on her neck, concomitant with worsened swallowing, and mild pain. The thyroid nodule appeared 13 years after diagnosis of sarcoma of the abdominal wall. 

Initial physical finding was on the neck palpable large, prominent, markedly firm, mildly painful resistance on right side, no fixation. Pemberton sign positive.

### 2.1. Differential Diagnosis

We considered several possibilities including metastasis from the known sarcoma or other frequently occurring metastases from carcinomas (renal, breast, gastrointestinal, lung, and melanoma) primary thyroid carcinoma, lymphoma, and benign lesions (adenoma, pseudocyst, and hemangioma). 

### 2.2. Results of Our Examinations

**Biochemistry**

TSH 0.363 mIU/l (N 0.32–5.0 mIU/l), fT4 14.6 pmol/l (N 9.0–19.0 pmol/l), aTG 1 U/ml (N < 1 U/ml), aTPO 1 U/ml (N < 1 U/ml), iPTH 6 pmol/l (N 1.48–8.43 pmol/l).

**US Thyroid**

Right lobe (RL) 50 ml, left lobe (LL) 7.3 ml, hypoechogenic nodule in middle LL 4 × 5 × 7 mm, all RL is filled with hypo- to anechogenic mass pressing the trachea to the left by 1 cm and compressing it to 20 mm (above goiter 34 mm). No pathological lymphatic nodes.

**FNAB : Micro**

Hemorrhagic background, disperse, isolated clusters of round, and oval pleomorphic nuclei of suspicious appearance were found. Conclusion: Needle Aspiration (FNA) Guidelines Committee IV [29] was category IV/3, that is, suspicious for other primary or secondary malignancies.

**CT Neck and Thorax**

Voluminous goiter right side, two metastases in lung.

**MRI Neck**

Nodular goiter right lobe on average 6x4x6 cm, without breakup, spreading retrotrachealy and compressing and deviating the trachea, and enlarged lymphatic nodes on neck were not found.

**Laryngoscopy**

Vocal cords freely movable.

### 2.3. Course and Diagnostic Result

The patient was scheduled for surgery following our examinations formechanical syndrome and FNAB suspicious malignant findings of dermatosarcoma metastasis. She underwent hemithyroidectomy (right lobe and isthmus with tissue volume of about 60 ml) without complication. 

### 2.4. Histology

Apparent angioinvasion and infiltration surrounding fat and muscle tunic tissue. Immunoprofile of tumorous cells, (vimentin strong positive, CD34 focal positive, and CD117 negative). Other helpful methods to differentiate mesenchymal versus epithelial lesions are staining of thyroid transcription factor-1 (TTF-1) and thyroglobulin.

**Figure 1:**
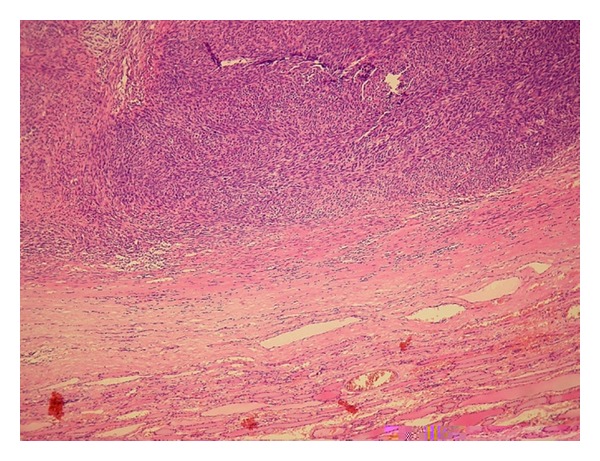
Hematoxylin-eosin staining, 100x, storiform, cellular organized tumor on periphery normal thyroid.

**Figure 2:**
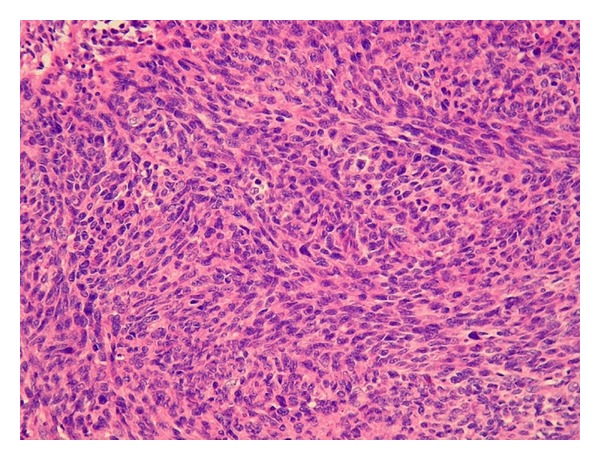
Hematoxylin-eosin staining, 400x, markedly tumorous, numerous polymorphs, tumorous cells.

**Figure 3:**
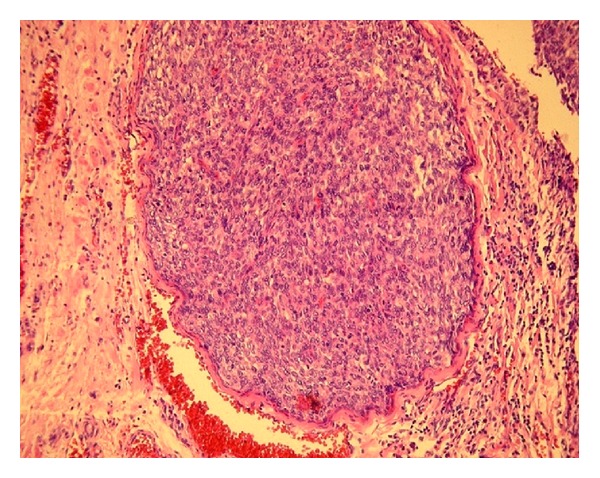
Hematoxylin-eosin staining, 200x, tumor angioinvasis.

Morphology (epitheloid, more spindle shaped) and marked mitotic activity is indicative for fibrosarcomatous variant dermatofibrosarcoma protuberans (Figures 1, 2, and 3). There was an agreement from local abdominal and thyroid histological finding as a metastasis from primary tumor to the thyroid. 

Oncologist supplemented examination c-kit (D 117) and considered treatment with imatinib (doxorubicin + ifosfamide) and radiotherapy. Secondarily after hemithyroidectomy were successfully removed bilateral lung—together four—metastases. Condition of the patient is very good.

## 3. Discussion

The thyroid gland is known but unusual site for metastases from sarcomas. There are 10 known cases of leiomyosarcoma metastases to the thyroid, 5 are from primary uterine leyomyosarcoma [22], while there is one case each of metastasis from histiosarcoma of the ankle [23], histiosarcoma of the thigh [24], carciosarcoma [25], liposarcoma of the thigh [26], leiomyosarcoma of the duodenum [27], and leiomyosarcoma of the leg [28]. Other possible and described metastases to the thyroid gland are shown in Table 1.

Clinically, patients tend to be in the their sixth decade of life or older, usually have a mass on the neck, along with dysphagia, hoarseness, and cold intolerance [23].

Diagnosis of a thyroid nodule as ametastatic lesion is not possible based on clinical and radiological grounds. FNAB is an easy, simple, and useful procedure.

Debulking or thyroidectomy is often indicated to confirm diagnosis and to alleviate any symptoms of neck compression [24].

After surgical management, the administration of systemic therapy is recommended [8].

The prognosis of these patients is grave, although surgical treatment prolongs the time of life (average 34 months with surgery versus 24 without) [12].

This case of dermatofibrosarcoma of the abdominal wall spreading to the thyroid gland is quite rare.

## 4. Conclusion

Metastasis should be considered in any patient with a previous history of malignancy and a new thyroid mass. Diagnosis is possible with FNAB and definitive after surgical treatment with histology. Chemo- and/or radiotherapy may be administered following surgery with various results. In some cases dermatofibrosarcoma protuberans may also be adequately treated with imatinib.

## Figures and Tables

**Table 1:** Metastatic diseases of the thyroid gland.

Primary tumor	References
Oral cavity carcinoma	[9]
Lingual squamous cell carcinoma	[10]
Parotid gland carcinoma	[9, 10]
Nasopharynx carcinoma	[9, 11]
Oropharynx carcinoma	[9]
Laryngeal carcinoma	[9]
Esophageal carcinoma	[9, 10, 12–14]
Cervical paragangliom	[15]
Gastric carcinoma	[11, 16]
Pancreatic carcinoma	[17]
Cholangiocarcinoma	[11, 18]
Hepatocellular carcinoma	[19]
Colon carcinoma	[13, 15]
Merckel cell carcinoma	[13, 15]
Breast carcinoma	[11, 14, 15, 20]
Kidney carcinoma	[12–14]
Bladder carcinoma	[13, 15]
Prostate carcinoma	[14]
Lung carcinoma	[11, 13–15, 20, 21]
Uterine carcinoma	[12–15]
Neuroendocrine tumors	[14, 21]
Malignant phylloides	[11]
Melanoma	[15]
Lymphoma	[14]
Sarcoma	[22–28]
